# Reply to Mazzaferro et al. and Niebaum: The limits of alignment: Grounding executive functions without assuming special contexts

**DOI:** 10.1073/pnas.2603106123

**Published:** 2026-04-06

**Authors:** Ivan Kroupin, Helen Elizabeth Davis, Emily Burdett, Joseph Henrich

**Affiliations:** ^a^Department of Comparative Cultural Psychology, Max Planck Institute for Evolutionary Anthropology, Deutscher Platz 6, Leipzig 04103, Germany; ^b^Department of Psychological and Behavioral Science, London School of Economics and Political Science, Clement House, 99–101 Aldwych, London WC2B 4JF, United Kingdom; ^c^Department of Human Evolutionary Biology, Harvard University, 11 Divinity Avenue, Cambridge, MA 02138; ^d^School of Human Evolution and Social Change, Arizona State University, 900 Cady Mall, Tempe, AZ 85287; ^e^Institute of Human Origins, Arizona State University, Institute of Human Origins, Arizona State University, 777 East University Drive, Tempe, AZ 85287; ^f^Department of Psychology, Nottingham University, School of Psychology, University of Nottingham, University Park, Nottingham NG7 2RD, United Kingdom

We thank the commentary authors for pointing out how our original dichotomy—between “executive functions” as universal and culturally specific—can be overcome, albeit by qualitatively expanding the scope of “executive function” as it is traditionally defined (e.g., refs. 1 and 2). In fact, this universal-and-culturally specific notion (“inclusive EFs”) stands to integrate our data with the crucial work of commentary authors in studying local patterns of EF engagement (3–5) and supporting development in a diverse array of contexts (6, 7). Such an inclusive approach, however, depends upon moving beyond the notion that tasks must be “aligned” with populations.

## Instrumental vs. Special Alignment

Mazzaferro et al. (8) argue that because “traditional executive function (EF) tasks developed in Western contexts do not validly assess EF in non-Western contexts” the contribution of our paper (9) is limited to further expanding “a large body of evidence suggesting that contextually misaligned tasks fail to capture how children engage EFs.”

We respectfully disagree. Two kinds of “alignment” are important to distinguish here. The first is instrumental—what Jukes et al. (7) summarize as “translating, adapting wording, and ensuring that culturally relevant examples are used” (p. 3). We control for these low-level issues in our study, and Mazzaferro et al. do not propose that such controls were insufficient.

Rather Mazzaferro et al. suggest that EFs in each population should be measured in a specific, culturally appropriate set of circumstances, i.e., special alignment. To illustrate this notion, consider two further, related examples. First, in developing EF measures for low- and middle-income countries, Obradović et al. (6) interpreted perseveration on Dimensional Change Card Sort [DCCS, (10)] as evidence that children “did not comprehend the task”—excluding this result from their main analyses and the task from their battery on the basis of not adequately reflecting EFs in their sample (Supporting Information, p. 7).

Second, Jukes et al. argue that accurate EF measurement “depends on participants’ willingness to adhere to and engage fully with task requirements” and that task contexts must be “familiar” or else risk participants “feeling uncomfortable and fostering...nonrepresentative performance” [(7), p. 4, 6-7].

## The Limits of Special Alignment

Special alignment can be difficult to reconcile with the reality of changing circumstances. Consider the case of a Himba child, primarily occupied with herding and other traditional activities. What tasks are relevant for understanding her EFs? Certainly specially aligned tasks are important for capturing regulation within her present context, and the alignment strategies discussed by Jukes et al. are invaluable in this case. However, if she is likely to attend school in future—as is increasingly the case for traditional groups—we are equally well-motivated to assess her management of demands overlapping with the unfamiliar context of formal schooling (e.g., in standard EF tasks). This is not because tasks assessing school-like demands are uniquely important or can serve as domain-general assessments of “EFs”—skills required for school-like tasks have their own limitations across diverse environments (e.g., refs. 11 and 12).

By the same token, special alignment risks inadvertently obscuring important data in ongoing work:

First, classifying cross-cultural differences in DCCS performance as simply a failure of measurement, in our data or that of Obradović et al., risks flattening or simplifying a potentially vital result. Given that inclusive EFs involve culturally evolved abilities relevant for regulation, the skills, and dispositions required for DCCS may well be part of “executive function”—i.e., executive function skills adapted to the particular cultural environments such as formal schooling. (The same logic applies equally to other tasks, see, e.g., discussion of Backwards Span in ref. 6, Supporting Information)

Niebaum’s (13) analysis provides empirical support for this idea—showing that performance on tasks which could have been ruled out as “unaligned” in fact correlates with performance on math and reading tests in our data. This result is consistent with an overlap in culture-specific skills for manipulating arbitrary rules and stimuli (as in, e.g., both mathematics and DCCS).

A similar limitation comes from using only tasks that are “comfortable” or “familiar” to participants and which adjust to their existing tendencies, e.g., in adhering (or not adhering) to rules. If “EFs” include culturally specific skills for regulating attention, why should we think that patterns of how individuals tend to adhere to rules and engage with tasks should be excluded from study? After all, the latter are almost certainly part of culturally evolved packages supporting regulation in particular environments such as schools (and likely having limited efficacy in others).

Similarly, why should we think that life outcomes will depend exclusively or primarily on EFs as they are deployed optimally in familiar, comfortable situations? This question is especially pressing in a world where regulation demands are changing rapidly—whether Himba enrolling in school or Boston children faced with environments increasingly saturated by technologies designed to capture attention.

## Inclusive EFs and Relevant Demands

Task choice, then, is not a question of “will it be aligned with current abilities and dispositions?” as per special alignment—but rather “does the task present a set of demands that overlap with the demands of current or future environments?” The latter question extends special-alignment approaches—from tasks whose demands overlap with participants’ prior experiences to task–population mappings more generally.

This general mapping suggests a research program capturing demands of environments predictably faced by different populations—and how our tasks do (or do not) reflect these demands. This approach dovetails with ongoing efforts to study local, contextualized EFs (3–5). Furthermore, it motivates applying tools from fields such as ethnography, social psychology, and cultural evolution to further map the space of regulatory demands across human environments (see, e.g., refs. 14 and 15 for related discussion). Studying universal EFs, on this account, requires tasks whose demands are demonstrably shared across human ecologies, rather than primarily culture-specific (as our data suggest is the case for traditional EF tasks).

In sum, moving beyond the logic of special alignment clarifies how a program of inclusive EFs integrates our work with the commentary authors’ valuable ongoing efforts, while also motivating complementary future directions.
